# Abnormal Driving Pattern Detection from GPS Trajectories Using Vision Transformer

**DOI:** 10.21203/rs.3.rs-8653475/v1

**Published:** 2026-02-06

**Authors:** Seyedeh Gol Ara Ghoreishi, Kwangsoo Yang

**Affiliations:** 1 Computer Science, Florida Atlantic University, Boca Raton, FL, USA.; 2 Computer Science, Florida Atlantic University, Boca Raton, FL, USA.

**Keywords:** Spatiotemporal data, GPS data, trajectory analysis, driving behavior, older driver classification, vision transformer, attention mechanism, deep learning

## Abstract

Given GPS points on a transportation network, the Driving Pattern Detection (DPD) problem aims to classify drivers as normal or abnormal based on their driving behavior. The DPD problem is challenging due to the variability in trip lengths, routes, and spatial patterns, which complicates input standardization for deep learning models. In this paper, we introduce a novel spatial representation learning framework for the DPD problem by analyzing driving patterns using a real-world dataset. We propose using binary grid images to capture the spatial structure of driving trajectories and present a new driving behavior representation for input to a Vision Transformer (ViT) model for driver classification. The experimental results demonstrate the effectiveness of the proposed algorithm, achieving an F1 score of 94% that significantly outperforms the baseline models. The results indicate that binary grid representations can effectively encode interpretable spatial patterns in driving behavior, with direct relevance to improved driver classification, road safety, and cognitive health assessment.

## 1 Introduction

Given GPS trajectory data, the Driving Pattern Detection (DPD) problem aims to distinguish between normal and abnormal drivers. This task is particularly important for enhancing road safety and enabling timely, targeted interventions for older drivers with Mild Cognitive Impairment (MCI). This problem is challenging because drivers’ pattern varies based on factors such as trip origin and destination, location, time, and the chosen routes. Moreover, deep learning models typically require inputs of fixed size, which presents a challenge when working with GPS trajectory data, as trips naturally vary in length and shape. Although some trips are long and others short, trip length or mileage alone does not reliably indicate meaningful behavioral insight and whether a driving pattern is abnormal. A common approach is to standardize trip lengths by dividing them into equal-sized segments. However, this can result in the loss of important contextual information and prevent analysis of the entire trip as a cohesive unit. Another traditional method is to analyze trips with the same origin and destination, focusing on the route shape. Yet, this is impractical, as drivers typically travel between different locations and do not follow consistent paths.

To address these challenges, we propose a novel method that converts raw GPS trajectories into fixed-size binary grid representations. This representation preserves the complete spatial structure of each trip while providing a standardized format suitable for deep learning-based classification.

**Fig. 1:**
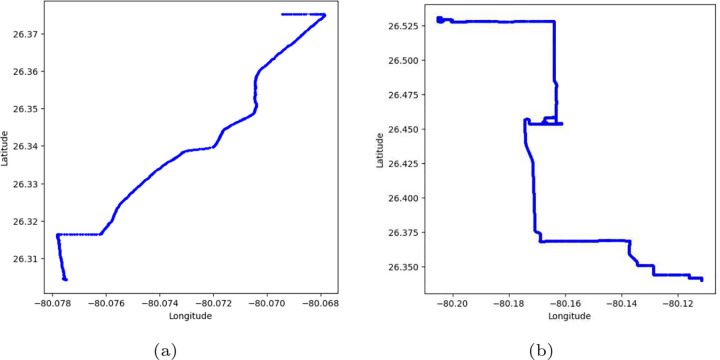
Trip data containing a range of Longitudes and Latitudes.

**Fig. 2:**
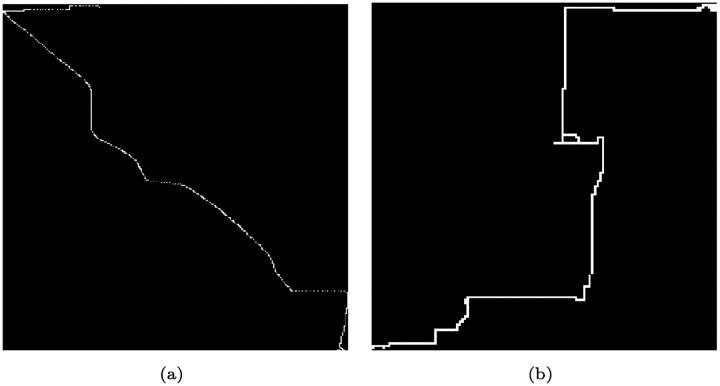
Grid representation of a driving trip. The GPS coordinates were converted into a 128×128 binary grid. This black-and-white image visualizes the spatial trajectory of the trip for analysis.

The conversion of trips into fixed-size binary grids enables the preservation of the overall shape and spatial structure of driving behavior, independent of trip length or the complexity of the road network. Figure 1 illustrates GPS trajectory data for a sample trip, while Figure 2 presents their corresponding fixed-size binary grid representation. By treating each grid as an image, we leverage Vision Transformers (ViTs) to extract both local and global spatial features effectively. ViTs are particularly well-suited for this task due to their capability to capture long-range dependencies via self-attention mechanisms. Our approach facilitates robust classification of trips as normal or abnormal without the need for fixed-length inputs or uniform routes, thereby offering strong adaptability to real-world driving scenarios.

## 2 Problem Formulation

Our study aims to develop a predictive model that utilizes an attention mechanism to detect abnormal driving behavior. The problem is formulated as follows:

- **Input**
  - A set of driving trips collected from in-vehicle sensors, where each trip consists of a sequence of latitude–longitude coordinates sampled over time,
  - A predefined grid cell size *c* for spatial discretization,
  - The minimum length of a trip *α*
- **Output**
  - A binary label for each trip indicating driving behavior (normal or abnormal).
- **Objective**
  - Maximize the performance of the classification to distinguish normal vs. abnormal trips based on spatial driving patterns.
- **Constraints**
  - The length of a trip > *α*.

## 3 Application Domain

This research lies at the intersection of transportation systems and machine learning, leveraging real-world driving behavior to support the early detection of cognitive decline in older adults. Driving is a cognitively demanding activity that requires the coordinated engagement of memory, attention, executive function, visuospatial processing, and rapid decision-making. As a result, even subtle cognitive disruptions, such as those associated with mild cognitive impairment (MCI), can manifest as measurable changes in everyday driving behavior.

Driving behavioral anomalies, such as inconsistent route choices, difficulty navigating familiar areas, and an increased incidence of missed turns, may signal emerging cognitive difficulties long before a formal diagnosis. While these deviations are often easily overlooked in clinical settings, they can be detected through continuous monitoring of GPS-based driving data. By modeling longitudinal driving behavior with advanced machine learning techniques, the proposed approach enables the extraction of behavior-level patterns indicative of MCI-related cognitive changes. This capability offers significant practical value for healthcare providers, caregivers, and transportation safety stakeholders by facilitating earlier identification of at-risk individuals and supporting data-driven clinical follow-up. Early detection may help reduce crash risk, inform individualized mobility planning, and support older adults in maintaining safe driving autonomy for as long as possible. From a broader perspective, this application contributes to safer transportation systems and scalable public health solutions by introducing a non-invasive, privacy-preserving framework for monitoring cognitive health through everyday mobility behaviors. The integration of telematics data with interpretable machine learning models offers a promising approach to enhancing cognitive wellness, promoting independence, and improving quality of life among aging populations.

## 4 Our Contribution

In this paper, we introduce a novel Transformer-based trajectory classification approach that efficiently models long-range dependencies and heterogeneous spatiotemporal patterns in real-world driving trajectories. Specifically, our contributions are as follows:

- We propose a trajectory-to-image encoding that converts GPS trajectories into binary spatial grids for efficient pattern learning.
- We design and implement a Vision Transformer (ViT) architecture for trip-level classification, exploiting self-attention to capture both local and global spatial dependencies in driving patterns.
- Our approach allows the model to handle trips of varying lengths and shapes without requiring trajectory segmentation or standardizing start/end points.
- We demonstrate that the proposed ViT-based framework outperforms traditional trajectory modeling approaches in identifying abnormal driving behavior, including patterns associated with early cognitive impairment.
- We validate the effectiveness of the proposed method on a large-scale, real-world dataset of older drivers, highlighting its applicability to public health monitoring and behavior-based risk assessment.

## 5 Literature Review

Research on detecting abnormal driving patterns has evolved from traditional statistical methods to deep learning-based trajectory analysis and, more recently, attention-driven models [1, 2]. This section reviews relevant studies in three main areas: (1) driving behavior analysis for cognitive impairment detection, (2) trajectory-based representations for machine learning, and (3) deep learning with Transformers for spatial pattern recognition.

5.1 Driving behavior analysis for cognitive impairment detection: Driving performance is closely related to cognitive abilities, as it requires attention, memory, and decision-making skills. Our previous research [3–7] has linked potentially abnormal driving behaviors, such as missed turns and repeated route and cyclic patterns loops to mild cognitive impairment (MCI) and early Alzheimer’s disease. Similar to our work, Huang[8] detected anomalous driving behaviors such as long detours and dense turn sequences. The method uses a higher-order Markov chain with a recursive Bayesian filter and applies a heading-change threshold to remove trivial movements and detect loops and cyclic driving patterns. Zhang et al. [9] detect detour behavior by analyzing trajectories with the same start and end points. They use Dynamic Time Warping (DTW) to measure similarity, accounting for temporal misalignments by calculating Euclidean distances between points.

Wang et al [10] proposed the Anomalous Trajectory Detection and Classification (ATDC) framework to identify anomalous driving patterns, including global detour, local detour, global shortcut, and local shortcut. Their approach first divides the city map into equal-sized grid cells and converts each trajectory into a sequence of grid cells. To measure trajectory similarity, they introduced the Difference and Intersection Set (DIS) distance metric, which computes an anomaly score for each trajectory.

5.2 Trajectory Representation for Machine Learning Trajectory data poses unique challenges for machine learning due to its variable length and irregular sampling. Traditional approaches often normalize trajectories by segmenting trips with similar origins and destinations. While effective for route clustering, such methods risk discarding critical behavioral information. For instance, Yoon and Lee [11] segmentize the trajectory data and create separate prediction models. Yu and Huang [12] used a variational autoencoder method that aggregates trajectory data and leverages in-vehicle monitoring systems to detect deviations from normal driving routes. Similarly, Zhang et al. [9] detect detour behavior by analyzing trajectories with the same start and end points. They use Dynamic Time Warping (DTW) to measure similarity, accounting for temporal misalignments by calculating Euclidean distances between points.

5.3 Deep Learning and Transformers for Trajectory Analysis Deep learning, particularly CNNs, has been widely applied to driving behavior recognition [13]. CNNs excel at extracting local spatial features; however, they have limitations in modeling long-range dependencies across complex trajectories. Transformers, originally introduced for natural language processing, have recently gained popularity in vision tasks due to their self-attention mechanism, which captures global context. Dosovitskiy et al. [14] showed that Vision Transformers (ViTs) outperform CNNs on various image classification benchmarks when sufficient data is available. In the driving domain, Huang et al. [15]applied attention-based models to trajectory prediction, demonstrating superior performance over traditional recurrent models in capturing global route dependencies. Jarndal et al. [16] indicated a Vision Transformer-based Driver Drowsiness Detection (ViT-DDD) system to classify a driver as drowsy or alert by analyzing full facial images extracted from video frames. Unlike earlier approaches that focused on specific facial features, this method leverages the global context of the entire face using Vision Transformers for more comprehensive and accurate classification. You et al. [17] introduced a GAF-ViT Transformation Module to convert multivariate driving behavior sequences into multi-channel images for driving behavior classification. Features such as speed, acceleration, and headway were each transformed into two Gramian Angular images and Gramian Angular Difference Field (GADF). These images were stacked and concatenated across all features to form a comprehensive multichannel image, enabling the application of Vision Transformers for accurate classification of different driving behaviors. Li et al. [18] encoded GPS points (latitude and longitude) into a one-dimensional index using a Hilbert curve and incorporated them into a multiscale spatially sensitive embedding module, which was then fed into a Transformer-based sequence modeling module for further processing. Mohammed et al. [19] proposed a lightweight Vision Transformer trained with pseudo-label-based semi-supervised learning to detect distracted driving with minimal labeled data. The approach uses a hybrid architecture that combines local convolution-like biases with global attention for feature extraction. Guo et al. [20] introduced a Binary Encoding (BE) representation to model trajectories for predictive tasks. In this approach, to process multidimensional trajectory data using a deep learning model, the method first converts continuous attributes (floats) to integer values and then encodes these integers as binary codes using the base-2 numeral system.

Our study explores spatial grid and image-based trajectory representations to preserve the overall shape and spatial context of trips. However, few studies have applied Transformer-based architectures to grid-encoded GPS trajectories for cognitive health assessment. Most existing approaches rely on handcrafted features or CNN-based models, which struggle to capture both global spatial dependencies and long-range relationships between GPS points across entire trips.

## 6 The proposed method of using Transformer on GPS trajectory data

### 6.1 Basic Concept

**Fig. 3:**
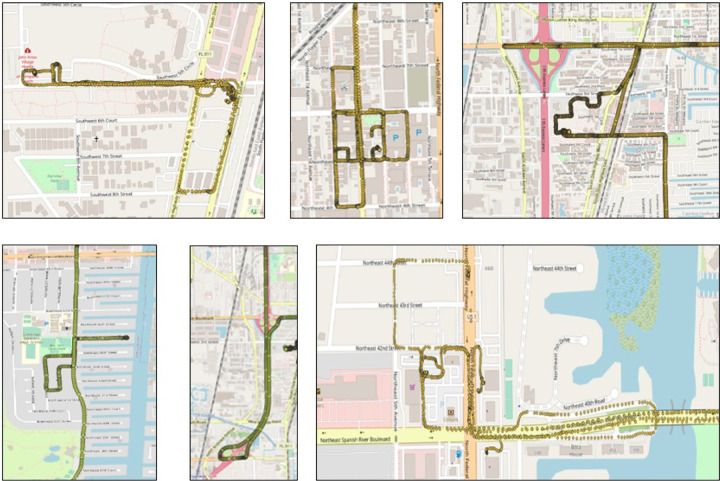
Snapshot of potentially abnormal driving behaviors, including cyclic patterns, and road repetitions, from the real-world dataset used in this study[4].

Abnormal driving behaviors are indicative of distraction, confusion, or cognitive decline. These include repeated path selections, cyclic driving patterns, and long-distance U-turns, which may indicate impaired navigation or difficulty adapting to unfamiliar routes. Figure 3 presents examples of such behaviors observed in our real-world driving dataset, which may be associated with early signs of cognitive impairment or distracted driving. The spatial characteristics of these behaviors are clearly manifested in the shape and structure of the travel trajectories. For example, cyclic routes may indicate disorientation or attempts to relocate a missed destination, while long, unnecessary U-turns may reflect confusion or inattentiveness. To analyze these behaviors effectively, the spatial structure of each driving trajectory must be preserved. Therefore, we adopt a trajectory-to-image transformation that enables direct recognition of spatial patterns.

### 6.2 Trajectory-to-Image Transformation for Deep Learning Models

Each driving trip is represented as a two-dimensional binary matrix (like a black-and-white image), where grid cells encode whether a particular area was visited during the trip. The transformation process includes the following steps:

1. Bounding Box Extraction:
  For each trajectory, the minimum and maximum latitude and longitude values are computed to define a bounding box:
min_lat,max_lat,min_lon,max_lon
2. Grid Discretization:
  The bounding box is divided into a fixed number of rows and columns, resulting in a binary grid of resolution *H* × *W* (e.g., 64 × 64). Each cell in the grid corresponds to a spatial region of equal size, with the resolution determining the spatial granularity.
3. Binary Encoding of Trajectory Points:
  Each GPS coordinate in the trajectory is assigned to its corresponding grid cell based on its geographic location. A cell is assigned a value of 1 if one or more GPS points fall within its bounds; otherwise, it is set to 0.
  Let *G* ∈ {0, 1}^*H*×*W*^ be a binary matrix representation of the grid, where *H* and *W* denote the number of rows and columns, respectively. Each element *G*_*i,j*_ indicates whether cell (*i, j*) was visited during the trip. Formally, the binary value *G*_*i,j*_ of cell (*i, j*) is defined as:
$$G_{i,j}=\left\{\begin{array}{ll} 1, & \text{if}\;\text{any}\;\text{GPS}\;\text{point}\;\text{lies}\;\text{in}\;\text{cell}(i,j)\\ 0, & \text{otherwise} \end{array}\right.$$
  This binary encoding transforms raw trajectory data into a fixed-size, imagelike grid, enabling the application of deep learning models designed for image classification.
4. Trip-Specific Normalization:
  The transformation is applied independently for each trip based on its local bounding box, rather than using global coordinates. This ensures that each binary grid captures the spatial characteristics of the trip with consistent resolution and scale, regardless of geographic location.

### 6.3 Dataset Construction

Once individual trips have been converted into binary grids, they are aggregated to form a structured dataset suitable for deep learning. Each sample in the dataset corresponds to one trip, represented as a 2D binary matrix. These matrices are stacked into a 3D tensor of shape:

N×H×W

where *N* is the total number of trips, and *H* × *W* is the fixed grid resolution. For compatibility with convolutional and transformer-based architectures that expect channel dimensions (e.g., grayscale images).

### 6.4 Handling Class Imbalance via Data Augmentation in Trajectory-to-Image Representation

**Fig. 4:**
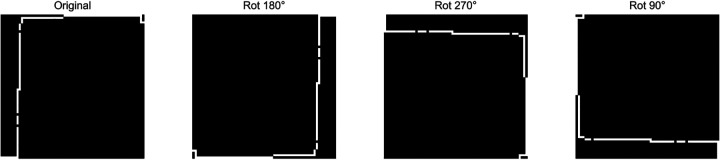
Original and rotated representations of a driving trip grid. The original binary grid (left) is shown alongside 180°, 270°, and 90° rotations.

Trajectory-to-Image transformation provides an effective strategy for addressing class imbalance through targeted data augmentation. In our approach, each trip is represented as a binary grid image, and we apply rotational transformations (e.g., 90°, 180°, and 270°) to augment samples from the minority class. Figure 4 illustrates this process by showing an original binary grid representation of a driving trip alongside its rotated versions, demonstrating how the same spatial trajectory can be viewed under different orientations while preserving its structural characteristics. These transformations preserve the spatial semantics of the trajectories while increasing sample diversity.

Data augmentation not only enhances the generalizability of deep learning models but also reduces overfitting, particularly in settings with limited or imbalanced data. While traditionally associated with convolutional neural networks, recent studies have shown that Transformer-based architectures, such as the Vision Transformer, can perform comparably or even better on image classification tasks when trained on large, diverse datasets. By applying augmentation selectively to the minority class, our method increases the model’s exposure to varied spatial patterns, mitigates bias toward the majority class, and improves the overall robustness and fairness of the classification process.

The trajectory-to-image transformation encodes each driving trip as a binary grid, enabling vision-based modeling of spatial and movement patterns. Using this representation, we apply a Vision Transformer (ViT) and compare it with CNN and ResNet baselines for abnormal driving detection. Preserving spatial structure and implicit temporal characteristics enhances detection accuracy and robustness.

### 6.5 Vision Transformer for Abnormal Driving Detection

Abnormal driving behaviors are not defined by isolated or unusual GPS points but by the overall spatial structure of a driving trip. Behaviors such as repeated routes, cyclic movement, and inefficient navigation emerge from how different areas of a trajectory relate to one another across space. Although driving trajectories are recorded as time-ordered latitude and longitude sequences, the analysis shifts focus from the precise timing of individual points to the spatial organization of the entire trip. Within this representation, temporal information is not modeled explicitly; instead, it is implicitly reflected through movement patterns, such as revisiting the same regions, redundant path traversal, or extended detours.

Vision Transformer (ViT) is well-suited for this type of analysis because it explicitly models relationships among spatial sub-regions using self-attention. The transformer processes grid-based trajectory representations by partitioning each image into fixed-size sub-regions, which are flattened and linearly embedded into a sequence of tokens. Positional embeddings are added to these tokens to preserve the spatial arrangement of sub-regions and maintain the geometric structure of the driving trip. The model then employs multi-head self-attention to capture interactions between all subregions simultaneously, enabling global spatial reasoning across the entire trajectory. This global attention mechanism enables the model to learn complex spatial configurations and long-range dependencies, which are essential for identifying abnormal driving behaviors, such as cyclic routes, repeated traversals, and inefficient path selection. By operating on structured spatial representations and modeling interactions among all regions without requiring explicit temporal alignment, ViT provides a robust framework for capturing complex spatial relationships in real-world driving data.

Table 1 presents the architecture of the ViT model used for grid-based driving trip classification. The model takes as input a single-channel binary grid of size 1×128×128, which represents the spatial footprint of an individual driving trip. This input is partitioned into non-overlapping sub-regions of size 8 × 8 through a convolutional patch embedding layer with a stride of 8, producing 256 patch embeddings. Each patch is linearly projected into a 128-dimensional embedding space, forming a sequence of patch tokens.

A learnable class token is prepended to the patch token sequence, and positional embeddings are incorporated to preserve the spatial arrangement of the patches, resulting in a total of 257 tokens. This token sequence is processed by a stack of six transformer encoder layers, each operating within the same 128-dimensional embedding space and utilizing GELU activation functions. Through multi-head self-attention, the transformer encoder captures global spatial dependencies across the trajectory grid, enabling effective modeling of long-range structural patterns.

Following the transformer encoder, layer normalization is applied to enhance training stability and convergence. The output corresponding to the class token is subsequently passed to a fully connected classification head, which maps the learned representation to two output classes corresponding to normal and abnormal driving behavior. A softmax activation function is applied to generate the final class probability distribution.

## 7 DATA COLLECTION

**Fig. 5:**
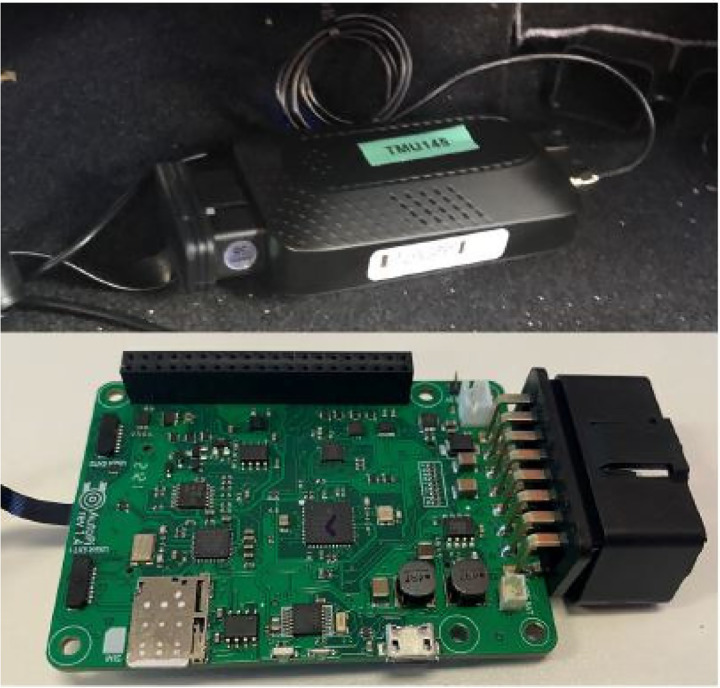
Raspberry-based TMU [7].

The team at Florida Atlantic University developed an in-vehicle sensing system that integrates telematics and vision sensing units to collect continuous, long-term naturalistic driving data [3–7, 21]. This system supports systematic analysis of real-world driving behavior, longitudinal driving patterns, and behavioral variability under everyday driving conditions. Figure 5 represents Telematics Measurement Units (TMUs) developed by AutoPi and built on the Raspberry Pi. Each TMU integrates a Global Positioning System (GPS) sensor, an inertial measurement unit (IMU) with tri-axial gyroscopes, an On-Board Diagnostics (OBD) interface, a 4G/LTE cellular modem, and onboard storage, enabling synchronized acquisition of spatial trajectories, vehicle dynamics, and operational signals. The TMUs continuously monitor vehicle power levels and implement energy-efficient sleep and hibernation modes when voltage falls below predefined thresholds, ensuring reliable long-term deployment with minimal power consumption [3–7, 21].

Using this sensing infrastructure, the study leveraged more than 72 million real-time trajectory records. The participant cohort consisted of older adult drivers aged 65 years and above, recruited through clinical and community outreach programs, and clinicians classified participants as cognitively normal or Mild Cognitive Impairment (MCI) based on comprehensive clinical evaluations[21]. Clinicians established ground truth labels for MCI through integrated assessments combining psychometric testing, standardized neuropsychological evaluations, and behavioral data analysis, and they further characterized cognitive status using Clinical Dementia Rating (CDR) scores alongside validated test batteries assessing memory, executive function, visuospatial ability, and language. The research team monitored cognitive changes at three-month intervals and employed a double-baseline assessment design to minimize practice effects, applying statistical corrections such as Reliable Change Indices (RCIs) to distinguish true cognitive decline from normal test–retest variability and ensure reliable longitudinal labeling [21]. All study procedures received approval from the Institutional Review Board (IRB), and the research strictly adhered to ethical guidelines for human-subject research, including participant privacy protection, data anonymization, and secure data handling [21].

## 8 EXPERIMENTAL EVALUATION

We conducted experiments to evaluate the performance of the proposed trajectory-to-image transformation with Vit, CNN and ResNet models. We aimed to answer two key questions: (1) What is the effect of data size? (2) What is the effect of the rotational data augmentation?

### 8.1 EXPERIMENT LAYOUT:

To mitigate potential biases and enhance the model’s robustness, we used a weighted F1 score and compared it with the Macro F1 score. To assess the generalizability of the proposed model, we conducted experiments on datasets of varying sizes (4 months and 2 years) to improve key performance metrics.

### 8.2 Evaluation Metrics:

The DPD problem is fundamentally a binary classification task. According to the real labels provided by the nursing college [21] and the predicted data labels, the possible outcomes are categorized as True Positive (TP), True Negative (TN), False Positive (FP), and False Negative (FN). To comprehensively assess model performance, we employed evaluation metrics including weighted F1-score and macro F1-score. The weighted F1-score was used to balance precision and recall across classes, with weights proportional to the number of samples in each class. In contrast, the macro F1-score treats all classes equally and is more sensitive to the minority class (MCI), reflecting the model’s ability to identify cognitively impaired drivers. The performance of the model was evaluated using the following metrics:

$$\text{Precision}=\frac{\text{TP}}{\text{TP}+\text{FP}}$$

$$\text{Recall}=\frac{\text{TP}}{\text{TP}+\text{FN}}$$

$$\text{F}1-\text{Score}=2\cdot \frac{\text{Precision}\cdot \text{Recall}}{\text{Precision}+\text{Recall}}$$

### 8.3 CNN Model

The grid-based input is processed by a CNN model to detect spatial dependencies. Table 2 provides an overview of the CNN layers. The model consists of three convolutional blocks, each comprising a 2D convolutional layer with a kernel size of 3×3, a ReLU activation, and 2×2 max pooling for spatial downsampling. The convolutional stack progressively increases the number of filters from 16 to 64, capturing hierarchical spatial features from the input grids. The extracted features are flattened and passed through two fully connected layers with 256 and 2 neurons, respectively, where the final layer performs binary classification using a softmax activation. The network was trained using the Adam optimizer with a learning rate of 0.001 and cross-entropy loss for 50 epochs. This baseline CNN serves as a reference for evaluating the performance advantages of the transformer-based architecture in modeling spatial dependencies within the proposed grid representation.

### 8.4 ResNet Model

To further evaluate the performance of the proposed trajectory-to-image approach, we compared the ViT model with a ResNet architecture of comparable depth. ResNet was selected due to its proven effectiveness in extracting hierarchical spatial features through residual connections, which helps mitigate the vanishing gradient problem and enhances feature propagation.

#### 8.4.1 Effect of Data Size

**Fig. 6:**
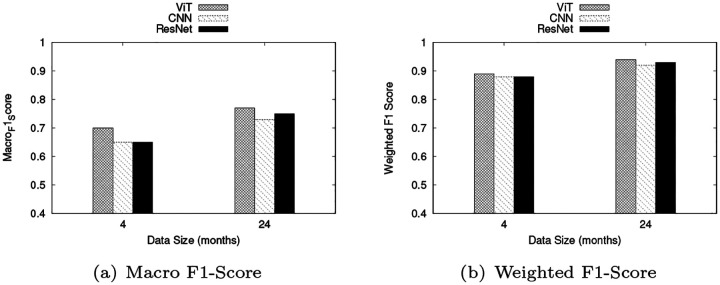
Effect of datasets within 4 months, and 2 years for macro and weighted F1Score across ViT, CNN, and ResNet models.

We conducted experiments using datasets spanning 4 months and 2 years to assess how varying data sizes influenced the performance of Vit, CNN, and RestNet models. Figure 6 illustrates the results for Weighted and macro F1-Scores across different data sizes.

#### 8.4.2 Effect of Data Augmentation

**Fig. 7:**
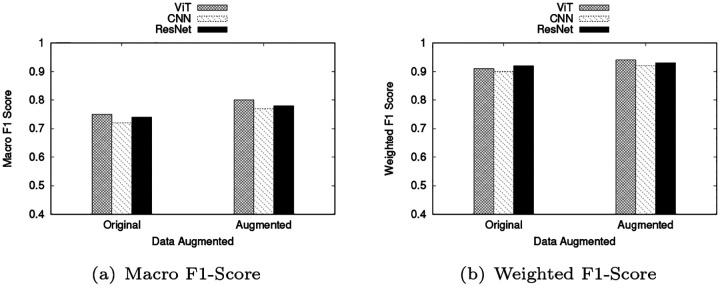
Effect of data augmentation for macro and weighted F1-Score across ViT, CNN and ResNet models.

To evaluate the robustness of the proposed grid-based representation and assess the influence of rotational variability, an ablation study was conducted using rotational data augmentation. The training grids were augmented by rotating them at 90 °, 180 °, and 270 °, effectively simulating spatial variations in driving trajectories while preserving the overall structure of the trip. This augmentation aimed to improve the model’s invariance to orientation and enhance generalization under diverse spatial configurations. Figure 7 summarizes the results of this experiment in the three baseline architectures: CNN, ResNet, and Vision Transformer (ViT). The inclusion of rotational augmentation consistently improved model performance, as measured by weighted and macro F1-Scores. The ViT model exhibited the most pronounced improvement, demonstrating its strong capability to capture rotation-invariant spatial dependencies. These results confirm that rotational augmentation enhances the representational robustness of the proposed grid-based dataset, particularly by benefiting transformer-based architectures that rely on global spatial context.

## 9 Discussion

Our study highlights significant improvements in detecting abnormal driving behavior by integrating a trajectory-to-image spatial representation with transformer-based architectures. This representation-driven approach enabled a systematic investigation of the robustness of the proposed framework through additional experiments on dataset scale and rotational data augmentation. The results demonstrate that increasing data volume leads to consistent performance gains, with the Vision Transformer benefiting most from larger datasets. Moreover, rotational augmentation significantly improves both macro and weighted F1-scores across all architectures, with the most pronounced gains observed for the Vision Transformer. These findings indicate that data augmentation enhances invariance to spatial orientation, improves sensitivity to minority classes, and strengthens generalization by exposing the model to a broader range of spatial configurations while preserving behavioral semantics.

These results highlight the potential of the proposed framework for real-world deployment in continuous driving behavior monitoring systems. The model’s ability to robustly detect abnormal driving patterns without trajectory segmentation or complex preprocessing supports scalable implementation in safety-critical applications. In particular, this approach offers a promising tool for enhancing road safety and enabling early behavioral risk assessment for older drivers with MCI or related cognitive decline, while remaining interpretable and clinically appropriate.

## 10 Conclusion

This paper presents a novel and effective framework for abnormal driving pattern detection by transforming GPS trajectories into fixed-size binary grid images and applying a Transformer model for driver classification. By preserving the full spatial structure of driving behavior and eliminating the need for trajectory segmentation or length normalization, the proposed method addresses key challenges in traditional trajectory modeling. Experimental results on a large-scale, real-world dataset of older drivers demonstrate that the ViT-based model significantly outperforms CNN and ResNet baselines, achieving high macro and weighted F1 Scores and demonstrating strong robustness to data imbalance and spatial variability. These results confirm that attention-based vision models are particularly well-suited for capturing global spatial dependencies in driving trajectories and identifying complex abnormal patterns indicative of cognitive or behavioral impairment. Beyond its technical contributions, this work has important implications for transportation safety and public health. The proposed approach provides a scalable, non-invasive, and privacy-aware mechanism for monitoring driving behavior and detecting early signs of abnormality associated with cognitive decline. By enabling earlier identification of at-risk drivers, the framework supports proactive clinical evaluation, informed mobility planning, and safer transportation systems.

Future research will focus on extending this framework to multimodal representations, longitudinal driver-level modeling, and real-time deployment scenarios. Overall, this study establishes a strong foundation for vision-based trajectory analysis and highlights the potential of transformer architectures to advance behavior-aware intelligent transportation and health monitoring systems.

## Figures and Tables

**Table 1:** Architecture of the Vision Transformer (ViT) model used for grid classification.

Module	Output Size	Patch / Kernel	Embedding / Units	Activation
Input Image	1 × 128 × 128	–	–	–
Patch Embedding (Conv2D)	256 × 128	8 × 8 / 8	128	Linear
Class Token + Position Embedding	257 × 128	–	128	–
Transformer Encoder ×6	257 × 128	–	128	GELU
Layer Normalization	257 × 128	–	–	–
Classification Head (FC)	2	–	128→2	Softmax

**Table 2:** Architecture of the CNN model used for grid classification.

Layer Type	Output Size	Kernel / Stride	Filters / Units	Activation
Input	1 × 128 × 128	–	–	–
Conv2D + ReLU	16 × 128 × 128	3 × 3 / 1	16	ReLU
MaxPooling2D	16 × 64 × 64	2 × 2 / 2	–	–
Conv2D + ReLU	32 × 64 × 64	3 × 3 / 1	32	ReLU
MaxPooling2D	32 × 32 × 32	2 × 2 / 2	–	–
Conv2D + ReLU	64 × 32 × 32	3 × 3 / 1	64	ReLU
MaxPooling2D	64 × 16 × 16	2 × 2 / 2	–	–
Flatten	16,384	–	–	–
Fully Connected (FC1)	256	–	256	ReLU
Fully Connected (FC2)	2	–	2	Softmax

**Table 3:** Architecture of the ResNet model used for grid classification.

Module	Output Size	Kernel / Stride	Channels / Units	Activation
Input Image	1 × 128 × 128	–	–	–
Conv2D + BN + ReLU	16 × 64 × 64	7 × 7 / 2	16	ReLU
MaxPooling2D	16 × 32 × 32	3 × 3 / 2	–	–
Residual Block ×2	16 × 32 × 32	3 × 3 / 1	16	ReLU
Residual Block ×2	32 × 16 × 16	3 × 3 / 2	32	ReLU
Residual Block ×2	64 × 8 × 8	3 × 3 / 2	64	ReLU
Residual Block ×2	128 × 4 × 4	3 × 3 / 2	128	ReLU
Global AvgPooling	128 × 1 × 1	–	–	–
Fully Connected (FC)	2	–	128→2	Softmax

## References

[1] Chen Y., Li X., Cong G., Bao Z., Long C., Liu Y., Chandran A.K., Ellison R.: Robust road network representation learning: When traffic patterns meet traveling semantics, 211–220 (2021) 10.1145/3459637.3482314

[2] Buchin M., Driemel A., Kreveld M.J., Sacristán V.: Segmenting trajectories: A framework and algorithms using spatiotemporal criteria. Journal of Spatial Information Science 3, 33–63 (2011) 10.5311/JOSIS.2011.3.63

[3] Ghoreishi S.G.A., Boateng C., Moshfeghi S., Jan M.T., Conniff J., Yang K., Jang J., Newman D., Furht B., Rosselli M., Tappen R., Jackson K.: Quadtree based driver classification using deep learning for mild cognitive impairment detection. IEEE Access (2025) 10.1109/ACCESS.2025.3539982 PMC12007693 40256415

[4] Ghoreishi S.G.A., Moshfeghi S., Jan M.T., Conniff J., Yang K., Jang J., Furht B., Tappen R., Newman D., Rosselli M., Zhai J.: Anomalous behavior detection in trajectory data of older drivers. In: Proc. IEEE 20th Int. Conf. Smart Communities, Improving Quality of Life Using AI, Robotics, and IoT (HONET), pp. 146–151 (2023). 10.1109/HONET59321.2023.10398771 PMC10985541 38567025

[5] Boateng C., Ghoreishi S.G.A., Yang K., Jan M.T., Tappen R.M., Jang J., Newman D., Moshfeghi S., Jackson K., Resnick R., Furht B., Rosselli M., Conniff J.: Spatial deep learning approach to older driver classification. IEEE Access 12, 191219–191230 (2024) 10.1109/ACCESS.2024.3425745 39748855 PMC11694628

[6] Jan M.T., Furht B., Moshfeghi S., Jang J., Ghoreishi S.G.A., Boateng C., Yang K., Conniff J., Rosselli M., Newman D., Tappen R.: Enhancing road safety: In-vehicle sensor analysis of cognitive impairment in older drivers. Multimedia Tools and Applications (2024) 10.1007/s11042-024-19192-1 PMC12252577 40657436

[7] Moshfeghi S., Jan M.T., Conniff J., Ghoreishi S.G.A., Jang J., Furht B., Yang K., Rosselli M., Newman D., Tappen R., Smith D.: In-vehicle sensing and data analysis for older drivers with mild cognitive impairment. In: Proc. IEEE 20th Int. Conf. Smart Communities, Improving Quality of Life Using AI, Robotics, and IoT (HONET), pp. 140–145 (2023). 10.1109/HONET59321.2023.10398768 PMC10982740 38562260

[8] Huang H.: Anomalous behavior detection in single-trajectory data. International Journal of Geographical Information Science 29(12), 2075–2094 (2015) 10.1080/13658816.2015.1063723

[9] Zhang H., Luo Y., Yu Q., Sun L., Li X., Sun Z.: A framework of abnormal behavior detection and classification based on big trajectory data for mobile networks. Security and Communication Networks 2020, 1–15 (2020) 10.1155/2020/8821702

[10] Wang J., Yuan Y., Ni T., Ma Y., Liu M., Xu G., Shen W.: Anomalous trajectory detection and classification based on difference and intersection set distance. IEEE Transactions on Vehicular Technology 69(3), 2487–2500 (2020)

[11] Yoon S., Lee K.: Aircraft trajectory prediction with inverted transformer. IEEE Access 13, 26318–26330 (2025) 10.1109/ACCESS.2025.3539742

[12] Yu W., Huang Q.: A deep encoder–decoder network for anomaly detection in driving trajectory behavior under spatio-temporal context. International Journal of Applied Earth Observation and Geoinformation 115, 103115 (2022) 10.1016/j.jag.2022.103115

[13] Zhang H., Luo Y., Yu Q., Sun L., Li X., Sun Z.: A framework of abnormal behavior detection and classification based on big trajectory data for mobile networks. Security and Communication Networks 2020(1), 8858444 (2020) 10.1155/2020/8858444

[14] Dosovitskiy A., Beyer L., Kolesnikov A., Weissenborn D., Zhai X., Unterthiner T., Dehghani M., Minderer M., Heigold G., Gelly S., Uszkoreit J., Houlsby N.: An image is worth 16×16 words: Transformers for image recognition at scale. arXiv preprint arXiv:2010.11929 (2020)

[15] Huang O., Rao H., Cai X., Wang T., Sun A., Xing S., Wu Y., Zhang B., Xu X., Liu S., Jia G.: Spatial temporal attention based target vehicle trajectory prediction for internet of vehicles. arXiv preprint arXiv:2501.00890 (2025)

[16] Jarndal A., Tawfik H., Siam A.I., Alsyouf I., Cheaitou A.: A real-time vision transformers-based system for enhanced driver drowsiness detection and vehicle safety. IEEE Access 13, 1790–1803 (2024) 10.1109/ACCESS.2024.3522111

[17] You J., Chen Y., Jiang Z., Liu Z., Huang Z., Ding Y., Ran B.: Exploring driving behavior for autonomous vehicles based on gramian angular field vision transformer. IEEE Transactions on Intelligent Transportation Systems 25(11), 17493–17504 (2024) 10.1109/TITS.2024.3445710

[18] Li S., Chen W., Yan B., Li Z., Zhu S., Yu Y.: Self-supervised contrastive representation learning for large-scale trajectories. Future Generation Computer Systems (2023) 10.1016/j.future.2023.05.019

[19] Mohammed A.A., Geng X., Wang J., Ali Z.: Driver distraction detection using semi-supervised lightweight vision transformer. Engineering Applications of Artificial Intelligence 129, 107618 (2024) 10.1016/j.engappai.2023.107618

[20] Guo D., Wu E.Q., Wu Y., Zhang J., Law R., Lin Y.: Flightbert: Binary encoding representation for flight trajectory prediction. IEEE Transactions on Intelligent Transportation Systems 24(2), 1828–1842 (2022) 10.1109/TITS.2022.3189168

[21] Tappen R., Newman D., Rosselli M., Jang J., Furht B., Yang K., Ghoreishi S.G.A., Zhai J., Conniff J., Jan M.T., Moshfeghi S., Panday S., Jackson K., Adonis-Rizzo M.: Study protocol for ‘in-vehicle sensors to detect changes in cognition of older drivers’. BMC Geriatrics 23(1), 854 (2023) 10.1186/s12877-023-04361-0 38097931 PMC10720160

